# Evaluation of the analytical performance of the MAGLUMI HEV IgM and IgG assays for automated detection of HEV antibodies and comparison with the microplate Wantai assay

**DOI:** 10.1186/s12985-026-03187-1

**Published:** 2026-05-08

**Authors:** Daying Geng, Yihua Zhu, Hongwei Zhang, Kun Liu, Shuguang Chen, Zhonggang Fang, Zhiqiang Zhang

**Affiliations:** 1 Shandong Public Health Clinical Center, Shandong University, Shandong, 250013 China; 2 Shanghai YangZhi Rehabilitation Hospital (Shanghai Sunshine Rehabilitation Center), School of Medicine, Tongji University, Shanghai, 201619 China; 3 Research & Development Department, Shenzhen New Industries Biomedical Engineering Co., Ltd. (Snibe), Shenzhen, 518122 China

**Keywords:** Hepatitis E virus, MAGLUMI, Chemiluminescence immunoassay, HEV IgM, HEV IgG, Diagnosis

## Abstract

**Background:**

Hepatitis E virus (HEV) infection screening and diagnosis are critical for controlling the HEV epidemic. Serological testing for anti-HEV IgM and IgG is widely used to diagnose acute and past HEV infections, respectively. This study aims to evaluate the analytical performances of the MAGLUMI HEV IgM and IgG assays for detection of HEV antibodies and comparison with the microplate Wantai assay.

**Methods:**

We assessed the precision, cross-reactivity, and interference of the MAGLUMI HEV IgM and IgG assays, as well as their agreement rates with the Wantai HEV IgM and IgG assays. Method comparison included in total 775 samples from 405 patients with suspected HEV infection and 86 asymptomatic blood donors. Among them, the HEV infection status of 136 individuals was confirmed through HEV RNA results, including 39 cases of acute infection, 3 cases of convalescence, and 94 cases of non-infected individuals.

**Results:**

The within-run and between-day imprecision for the MAGLUMI HEV IgM assay ranged from 1.54% to 3.71%, while for the MAGLUMI HEV IgG assay, it ranged from 0.00% to 4.35%. No cross-reactivity was observed in either assay. The positive and negative agreement rates between the MAGLUMI and Wantai IgM assay were 98.61% and 99.31%, respectively. The positive and negative agreement rates between the MAGLUMI and Wantai IgG assay were 94.95% and 97.20%, respectively.

**Conclusions:**

Overall, the analytical performance of the MAGLUMI HEV IgM and IgG assays is good, with reactivities comparable to those of the Wantai HEV IgM and IgG assays. However, due to the limited availability of HEV RNA detection results and lack of HEV genotype information, this study could not effectively evaluate the clinical performance of the reagent, and further studies with HEV RNA determination and genotyping are needed for a clinical validation.

**Supplementary Information:**

The online version contains supplementary material available at 10.1186/s12985-026-03187-1.

## Introduction

Hepatitis E virus (HEV) is predominantly transmitted via the fecal-oral route and is distributed globally. Its occurrence can manifest as either epidemic outbreaks or isolated sporadic cases. Large-scale outbreaks are more prevalent in areas characterized by inadequate sanitation and unsafe water sources. Human infections are chiefly attributed to genotypes 1 through 4. Specifically, genotypes 1 and 2 primarily infect humans, whereas genotypes 3 and 4 predominantly infect non-human mammals but can occasionally be transmitted to humans through the ingestion of undercooked meat or exposure to contaminated environments [1]. HEV infection induces an inflammation of the liver and can, in rare cases, lead to severe illnesses such as fulminant hepatitis (acute liver failure) [1]. In 2021, about 19.47 million new infections occurred, resulting in around 3450 deaths, accounting for 5.4% of global disability-adjusted life years (DALYs) related to acute hepatitis [1]. Although an effective vaccine for HEV infection exists, it is not yet widely used [2, 3]. While HEV infection is usually self-limiting, hospitalization is necessary for patients with fulminant hepatitis and symptomatic pregnant women due to the lack of specific treatment [4]. Clinically, HEV infection is indistinguishable from other forms of acute viral hepatitis, and definitive diagnosis typically relies on a combination of serology and nucleic acid testing (NAT) [5]. Serological testing detects anti-HEV immunoglobulin M (IgM) and G (IgG) antibodies in a person’s blood [6]. NAT is used to detect HEV RNA in blood or stool, which is especially important in regions where hepatitis E is rare and in immuno-suppressed patients with chronic HEV infection [5].

The currently available commercial serological HEV diagnostic tests primarily include ELISA, CLIA, and colloidal gold assays. However, the sensitivities and specificities of these tests vary widely. A recent meta-analysis encompassing 21 studies that compared the performance of anti-HEV assays with NAT tests found that the sensitivity of IgM tests ranged from 17% to 100%, while specificity ranged from 71% to 100% [7]. Similarly, the sensitivity of HEV IgG tests ranged from 15% to 100%, with specificity between 57% and 98% [7]. In recent years, several new automated anti-HEV assays have been brought to market, including Roche Elecsys Anti- HEV IgM and Anti- HEV IgG assays and LIAISON Murex Diasorin anti-HEV IgG and anti-HEV IgM assays [8, 9]. A recent study showed relative sensitivity and specificity of the Elecsys anti-HEV IgM assay were 98.6% and 98.9%, respectively compared to three commercial assays. For the Elecsys anti-HEV IgG assay, relative specificity was 81.2% and increased to 99.4% after neutralisation [8]. Another study evaluated the performance of four HEV antibodies detection methods and the results demonstrated the acute and convalescence phase sensitivity and specificity were 96.74% and 100%, respectively for LIAISON Murex Diasorin anti-HEV IgM assay. The sensitivity and specificity were 100% and 97.17%, respectively for LIAISON Murex Diasorin anti-HEV IgG assay [9]. It is important to note that fully automated assays remain limited and the ongoing development of highly sensitive and specific fully automated HEV antibody assays is still necessary.

The MAGLUMI HEV IgM and IgG assays (Snibe, Shenzhen, China) are two-step chemiluminescence immunoassays (CLIA) recently developed to detect IgM and IgG antibodies to HEV. The aim of this study is to evaluate their analytical performance and comparison with the microplate Wantai assay.

## Materials and methods

### HEV IgM and IgG assays

The MAGLUMI HEV IgM assay (Snibe Diagnostic, Shenzhen, China) employs a two-step capture chemiluminescence immunoassay methodology. Initially, the sample is mixed thoroughly with magnetic microbeads coated with anti-human IgM monoclonal antibodies and subjected to incubation. HEV IgM antibodies present within the sample bind to the anti-human IgM on the microbeads, resulting in the formation of an immune complex. Subsequently, following a washing step to remove unbound substances, ABEI-labeled HEV antigen (a combination of recombinant ORF2 and ORF3 antigens) is introduced and binds to the established complex. After an additional wash to eliminate any unbound reagents, Starter 1 and Starter 2 reagents are added to trigger a flash chemiluminescent reaction. The intensity of the emitted light is quantified by a photomultiplier as relative light units (RLUs).

Similarly, the MAGLUMI HEV IgG assay (Snibe Diagnostic, Shenzhen, China) utilizes a two-step indirect chemiluminescence immunoassay approach. In the first phase, the sample is thoroughly mixed and incubated with magnetic microbeads coated with HEV antigen (a combination of recombinant ORF2 and ORF3 antigens). HEV IgG antibodies present in the sample bind to the HEV antigen, forming a complex. Following a washing step, ABEI-labeled anti-human IgG monoclonal antibodies are added, which bind to the complex. After a subsequent wash to remove unbound components, Starter 1 and Starter 2 reagents are applied to initiate a flash chemiluminescent reaction. The emitted light is measured by a photomultiplier as RLUs. All samples analyzed in this study were assessed using the HEV IgM or IgG assays on the Snibe automatic immunoassay analyzer MAGLUMI X8, in strict accordance with the manufacturer’s instructions.

The Wantai HEV IgM and IgG EIA kits (Wantai Biologic Pharmacy Enterprise, Beijing, China) were used as comparator assays. Recombinant HEV pORF2 antigens were used in Wantai HEV assays.

### Quality control materials and clinical samples

The analytical precision of the two MAGLUMI HEV assays was assessed utilizing serum specimens in accordance with the Clinical and Laboratory Standards Institute (CLSI) EP15-A3 guidelines.

**Fig. 1:**
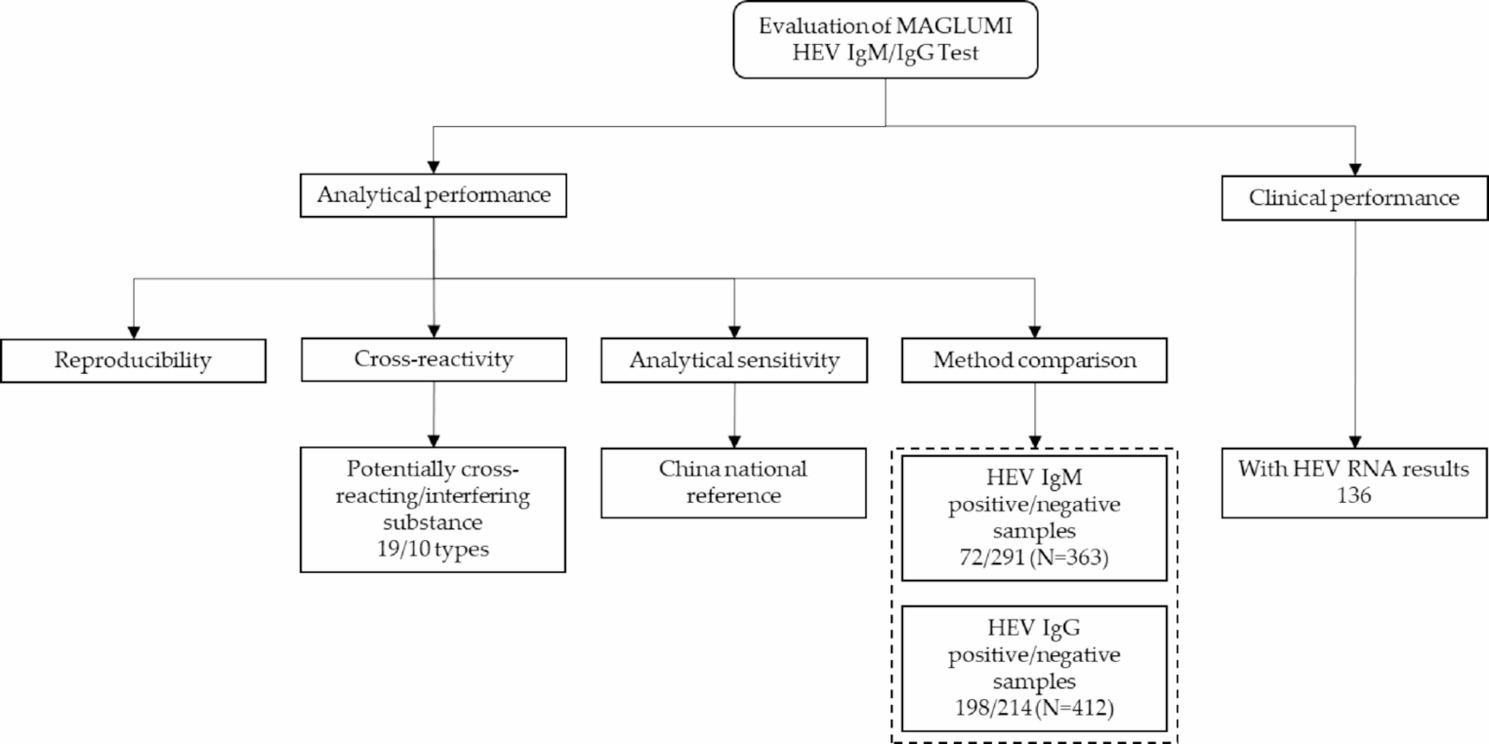
Study flow diagram

This prospective, cross-sectional observational study involved the consecutive collection of 603 serum or plasma samples from 405 patients with suspected HEV infection at two medical centers between November 2022 and November 2023, encompassing both inpatient and routine outpatient settings. Another 172 serum samples from 86 blood donors were consecutively collected at Shenzhen Blood Center in May 2025. Samples were preserved at ₋20℃ and thoroughly thawed prior to testing. All specimens underwent evaluation using the MAGLUMI HEV IgM and IgG assays, with reactivity defined as results equal to or exceeding 1.00 AU/mL. Comparative analysis was conducted between the MAGLUMI HEV IgM and IgG assay outcomes and those obtained from the Wantai HEV IgM and IgG assays. Additionally, the HEV infection status of 136 samples was confirmed via HEV RNA testing, serving as a reference for assessing the clinical performance of the MAGLUMI HEV IgM assay (Fig. 1).

### Endogenous sample interference

In total 179 and 123 samples containing potentially cross-reactive substances including samples generated from patients with infectious diseases (HAV, HBV, HCV, CMV, anti-HEV IgG or IgM, EBV, HSV, *Toxoplasma gondii*, rubella, syphilis, HIV), dialysis patients and rheumatic factor positive patients were evaluated with the MAGLUMI HEV IgM and IgG assays, respectively (Table 2). Ten types of interfering substances, including bilirubin (40.0 mg/dL), intralipid (1.0 g/dL), hemoglobin (2.0 g/dL), rheumatic factor (1500 IU/mL), anti-nuclear antibodies (398 AU/mL), human anti-mouse antibody (40 ng/mL), biotin (50 µg/mL), rapamycin (1.2 µg/mL), everolimus (6 mg/mL), sofosbuvir (2 µg/mL) were assessed with the MAGLUMI HEV IgM and IgG assays (Table 3). The results obtained after the addition of interfering substances were compared to those obtained following the addition of the corresponding solvents alone (e.g., distilled water and DMSO) to determine the interference rate.

### Analytical sensitivity using the China national reference

Due to the unavailability of the WHO standard for HEV antibodies (NIBSC Code: 95/584), this study utilized the China national reference panels for HEV IgM (Lot: 300011-20130601) and HEV IgG (Lot: 300014-20130601), which are calibrated against the WHO reference reagent (REFERENCE REAGENT FOR HEPATITIS E VIRUS ANTIBODY, human serum, NIBSC code: 95/584). The China national reference panels for HEV IgM and HEV IgG consist of 34 samples (20 negative reference materials, 12 positive reference materials, 1 LoD reference material and 1 precision reference material) and 42 samples (30 negative reference materials, 10 positive reference materials, 1 LoD reference material and 1 precision reference material), respectively. These panels were employed to evaluate the MAGLUMI HEV IgM and IgG assays in a standardized manner, following the manufacturer’s instructions (Fig. 1). The correlation between the assay-measured values for HEV IgM and IgG assays and the serial twofold dilutions of the sensitive reference materials was analyzed.

### Statistical analysis

Proportions and their corresponding Wilson 95% confidence intervals were performed using Microsoft Office Excel^®^ 2013. Variability was expressed as either the standard deviation (SD) or the coefficient of variation (CV).

### Ethics

The clinical performance evaluation was carried out in accordance with the ethical principles laid down in the Declaration of Helsinki. All samples from Mengchao Hepatobiliary Hospital of Fujian Medical University were residual specimens collected under a general ethical approval, with subjects having signed broad informed consent. Similarly, the samples from Shandong Public Health Clinical Centre were residual specimens; patient consent was waived because exempting subject from obtaining informed consent would not have a negative impact on the subject’s rights and interests, and human materials or data used in this study were anonymized, making it impossible to identify the subjects, and the research did not involve personal privacy concerns. Ethical approval was obtained from the Ethics Committee of Mengchao Hepatobiliary Hospital of Fujian Medical University (2022_062_01 and 2022_061_01) and the Ethics Committee of Shandong Public Health Clinical Centre (GWLCZXEC2022-136 and GWLCZXEC2022-137).

## Results

### Precision of the MAGLUMI HEV IgM and IgG assays

Within-run reproducibility was defined as the precision within-lot, within-run and within-instrument. Between-run reproducibility encompassed all variable factors, including run, day, and lot. The low and medium concentration samples, as well as quality control (QC) sample provided by the manufacturer were used to evaluate the precision of the MAGLUMI HEV IgM and IgG assays. The within-run reproducibility of the MAGLUMI HEV IgM assay yielded a CV ranging from 1.54% to 3.71%. Between-day reproducibility showed a CV of 1.98% to 2.86% (Table 1, Supplementary Table S1). For the MAGLUMI HEV IgG assay, within-run reproducibility produced a CV of 2.75% to 4.35%, while between-day reproducibility ranged from 0.00% to 3.02% (Table 1, Supplementary Table S2).

**Table 1:** Precision of MAGLUMI HEV IgM and IgG CLIA assays

				Within-Run		Between-Day	
		Total number of measurements	Mean (AU/mL)	SD (AU/mL)	%CV	SD (AU/mL)	%CV
HEV IgM	low	25	1.363	0.021	1.54	0.039	2.86
	medium	25	3.889	0.071	1.83	0.077	1.98
	QC	25	3.504	0.130	3.71	0.071	2.03
HEV IgG	low	25	1.816	0.050	2.75	0.000	0.00
	medium	25	10.097	0.367	3.63	0.122	1.21
	QC	25	3.906	0.170	4.35	0.118	3.02

AU, arbitrary unit; SD, standard deviation; CV, coefficient of variation; QC, quality control

### Cross-reactivity of the MAGLUMI HEV IgM and IgG assays

The cross-reactivity of the MAGLUMI assays was primarily evaluated by assessing potential interference from other infections prevalent in similar regions. All 179 and 123 samples containing potentially cross-reactive substances were non-reactive with the MAGLUMI HEV IgM and IgG assays, respectively (Table 2). Ten types of interfering substances, including bilirubin, intralipid, and hemoglobin, were assessed and were also non-reactive with the MAGLUMI HEV IgM and IgG assays (Table 3).

**Table 2:** Analytical specificity of MAGLUMI HEV IgM and IgG in samples containing potentially cross-reactive substances

Cross-reactive substances	IgM (*n*/*N*)	IgG (*n*/*N*)
Anti-HAV IgM positive	0/6	0/2
Anti-HBs positive	0/5	0/7
HBsAg positive	0/10	0/10
Anti-HBe positive	0/10	0/10
HBeAg positive	0/7	0/6
Anti-HBc positive	0/10	0/10
Anti-HCV positive	0/16	0/2
Anti-HEV IgG positive	0/10	-
Anti-HEV IgM positive	-	0/2
Anti-CMV IgM positive	0/21	0/20
Anti-CMV IgG positive	0/11	0/4
Anti-EBV VCA IgM positive	0/24	0/21
Anti-HSV-1/2 IgM positive	0/1	0/1
Anti-Toxo IgG positive	0/3	0/4
Anti-Toxo IgM positive	0/1	0/2
Anti-Rubella IgM positive	0/1	0/2
Anti-Syphilis positive	0/6	0/7
Anti-HIV positive	0/15	0/5
Dialysis insufficiency (dialysis patients)	0/5	0/5
RF positive	0/17	0/3
Total	0/179	0/123
Analytical specificity	100.00%	100.00%
95% CI	97.90%-100.00%	96.97%-100.00%

HEV, hepatitis E virus; HAV, hepatitis A virus; HBs, HBsAg; HBsAg, hepatitis B surface antigen; HBe, HBeAg; HBeAg, hepatitis B e antigen; HBc, hepatitis B core antigen; HCV, hepatitis C virus; CMV, cytomegalovirus; EBV, Epstein-Barr virus; HSV, herpes simplex virus; Toxo, *Toxoplasma gondii*; HIV, human immunodeficiency virus; RF, rheumatic factor

**Table 3:** Analytical specificity of MAGLUMI HEV IgM and IgG in samples containing interfering sub-stances

Interferent	Interferent concentration	HEV IgM (AU/mL)		Interference	HEV IgG (AU/mL)		Interference
		Solvent control	Interferent added		Solvent control	Interferent added	
Bilirubin	40.0 mg/dL	9.582	9.406	-1.84%	2.88	2.88	0.00%
		79.294	77.880	-1.78%	27.2	26.4	-2.94%
Intralipid	1.0 g/dL	9.062	9.791	8.04%	2.70	2.69	-0.37%
		75.831	79.661	5.05%	26.7	26.4	-1.12%
Hemoglobin	2.0 g/dL	9.062	9.017	-0.50%	3.13	3.09	-1.28%
		75.831	74.311	-2.00%	26.7	26.1	-2.25%
RF	1500 IU/mL	9.062	9.383	3.54%	3.14	3.01	-4.14%
		75.831	78.351	3.32%	27.5	25.2	-8.36%
ANA	398 AU/mL	9.062	9.055	-0.08%	3.13	2.88	-7.99%
		75.831	74.477	-1.79%	27.6	27.2	-1.45%
HAMA	40 ng/mL	9.062	9.157	1.05%	3.02	2.77	-8.28%
		75.831	74.950	-1.16%	27.7	27.5	-0.72%
Biotin	50 µg/mL	9.062	9.042	-0.22%	2.71	2.55	-5.90%
		75.831	74.289	-2.03%	26.7	26.7	0.00%
Rapamycin	1.2 µg/mL	10.489	11.162	6.42%	2.89	2.89	0.00%
		77.339	82.361	6.49%	26.2	26.2	0.00%
Everolimus	6 mg/mL	10.489	10.836	3.31%	2.92	2.84	-2.74%
		77.339	75.677	-2.15%	27.7	27.6	-0.36%
Sofosbuvir	2 µg/mL	10.489	10.380	-1.04%	2.87	2.89	0.70%
		77.339	80.921	4.63%	27.0	27.5	1.85%

Original concentration of HEV IgM (Medium: 9.665 pg/mL; High: 82.376 pg/mL) and HEV IgG (Medium: 3.08 pg/mL; High: 27.2 pg/mL) positive samples

HEV, hepatitis E virus; AU, arbitrary unit; RF, rheumatic factor; ANA, anti-nuclear antibodies; HAMA, human anti-mouse antibody

### Analytical sensitivity of the MAGLUMI HEV IgM and IgG assays

Both the MAGLUMI and Wantai HEV IgM and IgG assays detected the China national reference panels with an acceptable analytical sensitivity (Table 4). In this evaluation, the analytical sensitivity of both HEV IgM assays was ≥ 1:32 dilution, which complies with the common technical specification (CTS) requirement of ≥ 1:8 dilution. The analytical sensitivity of the MAGLUMI HEV IgG assay was 0.375 U/mL, meeting the CTS criterion of ≤ 3.0 U/mL (Table 4).

**Table 4:** Analytical sensitivity of the national reference for HEV IgM and IgG antibodies obtained using the MAGLUMI HEV IgM and IgG assays

National reference	Measure	Acceptance criteria	MAGLUMI HEV IgM and IgG
HEV IgM	Negative coincidence rate	100% (20/20)	100% (20/20)
	Positive coincidence rate	100% (12/12)	100% (12/12)
	LoD reference materials	LoD ≥ 1:8 (dilution)	LoD ≥ 1:32 (dilution)
	Precision reference materials	CV ≤ 15%	CV = 2.665%
HEV IgG	Negative coincidence rate	≥ 96.7% (29/30)	100% (30/30)
	Positive coincidence rate	≥ 90.0% (9/10)	100% (10/10)
	LoD reference materials	LoD ≤ 3.0 U/mL	LoD ≤ 0.375 U/mL
	Precision reference materials	CV ≤ 15%	CV = 1.721%

HEV, hepatitis E virus; LoD, Limit of Detection

### Method comparison of the MAGLUMI and Wantai HEV assays

The patient characteristics of the study are summarized in Supplementary Table S3. All 405 suspected HEV infected patients from medical centers have at least one sign or symptom of liver disease including yellow sclera, staining of the skin, dark-colored urine, jaundice, etc. (Supplementary Table S3). The demographic characteristics of the 86 asymptomatic blood donors were unavailable. Among these individuals, HEV RNA results confirmed that 39 patients were in the acute stage, 3 patients were in the convalescent stage and 94 individuals were uninfected. The infection status of other individuals is unknown (Supplementary Table S3). In total 363 samples (291 for Wantai HEV IgM negative) including 277 samples from patients and 86 samples from blood donors were used to evaluate method comparison of the two HEV IgM assays. Of the 363 samples, 71 tested positive on both the MAGLUMI and Wantai HEV IgM assays (Table 5). One sample tested positive on the Wantai assay but negative on the MAGLUMI assay (positive agreement: 98.61%; 95% CI: 92.54–99.75%) (Supplementary Table S4). Conversely, two samples tested positive on the MAGLUMI assay but negative on the Wantai assay (negative agreement: 99.31%; 95% CI: 97.52–99.81%) (Supplementary Table S4). This resulted in a total agreement rate of 99.17% (95% CI: 97.59–99.72%) between the two HEV IgM assays. Among the three discrepant samples, only one was tested for HEV RNA, which was negative. This sample tested positive for IgM with Wantai but negative with MAGLUMI. In total 412 samples (214 for Wantai HEV IgG negative) including 326 samples from patients and 86 samples from blood donors were used for the comparison of the two HEV IgG assays. 188 of the 412 samples tested positive on both the MAGLUMI and Wantai HEV IgG assays (Table 6). Ten samples tested positive on the Wantai assay but negative on the MAGLUMI assay (positive agreement: 94.95%; 95% CI: 90.95–97.23%) (Supplementary Table S5). Conversely, six samples tested positive on the MAGLUMI assay but negative on the Wantai (negative agreement: 97.20%; 95% CI: 94.02–98.71%) (Supplementary Table S5). This resulted in a total agreement rate of 96.12% (95% CI: 93.79–97.60%) between the two HEV IgG assays.

**Table 5:** Agreement between MAGLUMI and Wantai HEV IgM assays

MAGLUMI HEV IgM	Wantai HEV IgM	
	Positive	Negative
Positive	71	2
Negative	1	289
Positive agreement	98.61% (95%CI: 92.54–99.75%)	
Negative agreement	99.31% (95%CI: 97.52–99.81%)	
Total agreement rate	99.17% (95%CI: 97.59–99.72%)	

HEV, hepatitis E virus; CI, Confidence Interval

**Table 6:** Agreement between MAGLUMI and Wantai HEV IgG assays

MAGLUMI HEV IgG	Wantai HEV IgG	
	Positive	Negative
Positive	188	6
Negative	10	208
Positive agreement	94.95% (95%CI: 90.95–97.23%)	
Negative agreement	97.20% (95%CI: 94.02–98.71%)	
Total agreement rate	96.12% (95%CI: 93.79–97.60%)	

HEV, hepatitis E virus; CI, Confidence Interval

### Clinical performances of the MAGLUMI and Wantai HEV IgM assays

136 samples tested with HEV RNA to confirm their hepatitis E infection status, and most of them (97 out of 136) were HEV RNA negative, including 3 samples tested positive with both the MAGLUMI and Wantai HEV IgM assays. The rest 39 HEV RNA positive samples also tested positive with both HEV IgM assays (Supplementary Table S6).

## Discussion

This article reports the analytical performance of the MAGLUMI HEV IgM and IgG assays designed for use on the MAGLUMI series fully automated chemiluminescence immunoassay analyzer. The results demonstrated that the MAGLUMI HEV IgM and IgG assays exhibit good precision and analytical sensitivity, as evaluated by assessing factors such as cross-reactivity, interfering substances, and national reference standards. Additionally, when compared with the Wantai HEV IgM and IgG ELISA assays, the MAGLUMI HEV IgM and IgG showed negative agreement rate of 99.31% (289/291) and 97.20% (208/214), and positive agreement rate of 98.61% (71/72) and 94.95% (188/198), respectively, in samples from hospitalized patients and blood donors. Based on these results, it can be concluded that the MAGLUMI HEV IgM and IgG assays possess reasonably good reactivities comparable to those of the Wantai HEV IgM and IgG assays.

For the serological diagnosis of HEV, most available assays are manual ELISAs; however, several fully automated assays have recently become available [8–13]. Due to the variability in sensitivity and specificity among these assays, we selected the Wantai HEV IgM and IgG ELISA assays as comparator assays in this study because of their widely recognized performance [7, 14–16]. One limitation of this study was the absence of confirmation of antibody status using the reference standard immunoblot [6], which prevented determination of the true antibody status in discrepant samples. HEV RNA testing is not routinely performed in hospitals; only samples from 50 patients with a high suspicion of HEV infection were sent for RNA testing. Of these 39 tested RNA positive, while 11 tested RNA negative including 8 with IgM negative results. All RNA positive and 3 RNA negative samples tested positive with both MAGLUMI and Wantai IgM assays. Additionally, 94 samples that were IgM and RNA negative also tested negative with the MAGLUMI IgM assay. Due to the limited availability of RNA detection results, this study could not effectively evaluate the clinical performance of the reagent, and further studies are warranted.

According to previous studies on HEV prevalence in China, the most common genotype is genotype 4, followed by genotypes 1 and 3 to a lesser extent [17–19]. Previous studies have shown no sensitivity issues with Wantai HEV assays in detecting genotypes 3 and 4, despite the pORF2 antigens used in these assays being derived solely from a genotype 1 strain [20, 21]. A combination of recombinant ORF2 and ORF3 antigens, which used by the MAGLUMI assays may enhance their sensitivity [22]. However, HEV genotype information was lacking in this study, and it would be valuable to evaluate the sensitivity and specificity of the MAGLUMI assays using samples with known HEV genotypes.

The MAGLUMI HEV IgM and IgG assays are both chemiluminescence immunoassays (CLIA). CLIA technology has advanced significantly over the past several decades and is widely used in the diagnosis of infectious diseases including hepatitis viruses, HIV, and syphilis [23–26]. Due to its superior sensitivity, speed, and automation, CLIA is gradually replacing the EIA method in some high-volume clinical laboratories. The evolution of HEV antibody detection methods exemplifies this trend. It is worth noting that the MAGLUMI HEV IgM and IgG assays with similar detection fees can obtain results faster than Wantai HEV assays (around 30 min versus 90–120 min). NAT testing is recommended by the European Association for the Study of the Liver (EASL) to be used in combination with serology testing [6], however, it is challenging to initially screen for HEV infection using NAT testing due to its high cost and the need for specialized laboratory facilities.

In this study, we evaluated 16 types of potential interference from other infections, including cytomegalovirus (CMV) and Epstein-Barr virus (EBV), which have been reported to cause a high degree of cross-reactivity in HEV IgM detection [27–30]. We found that the MAGLUMI HEV IgM and IgG assays yielded negative results when testing HEV antibody-negative samples that were positive for anti-CMV IgM or anti-EBV VCA IgM. However, given the limited sample size, further evaluation is necessary to confirm that the MAGLUMI HEV IgM assay can reliably avoid false-positive anti-HEV IgM results in acute EBV and CMV infections.

Anti-HEV antibodies are often undetectable in immunocompromised individuals infected with HEV or in patients with chronic hepatitis E due to delayed or absent serological responses [31]. Therefore, HEV RNA testing is recommended for accurate diagnosis in these cases [6]. One limitation of this study is that we did not evaluate performance of the MAGLUMI HEV IgM and IgG assays in samples from immunocompromised or chronically HEV infected patients, and further studies are needed.

The Global Health Sector Strategies (GHSS) set the ambitious goal of ending the prevalence of viral hepatitis by 2030 [32]. To achieve this, it is essential to scale up viral hepatitis prevention, testing, and treatment to reduce liver disease. Testing for HEV infection is fundamental to these objectives. The MAGLUMI HEV IgM and IgG assays demonstrated reactivity comparable to Wantai, however, assessment of their diagnostic accuracy was lacked in this study, which is essential to support their application in the prevention and control of hepatitis E.

## Supplementary Information

Below is the link to the electronic supplementary material.

Supplementary Material 1.

Supplementary Material 2.

Supplementary Material 3.

Supplementary Material 4.

Supplementary Material 5.

Supplementary Material 6.

## Data Availability

No datasets were generated or analysed during the current study.
