# Supplementary material for: Evaluation of the analytical performance of the MAGLUMI HEV IgM and IgG assays for automated detection of HEV antibodies and comparison with the microplate Wantai assay
Source: Virol J. 2026 May 8;23:167. doi: 10.1186/s12985-026-03187-1 (PMC13321755; doi:10.1186/s12985-026-03187-1)
Supplement: Supplementary file 1 — Supplementary Material 1. [file 12985_2026_3187_MOESM1_ESM.docx]

| Days | Replicates | Test Results | | |
| --- | --- | --- | --- | --- |
|  |  | Sample 1  ( low concentration) | Sample 2  ( medium concentration) | Sample 3  (QC) |
| Day 1 | 1 | 1.39 | 3.98 | 3.71 |
|  | 2 | 1.41 | 3.97 | 3.53 |
|  | 3 | 1.4 | 3.9 | 3.49 |
|  | 4 | 1.41 | 3.93 | 3.51 |
|  | 5 | 1.37 | 3.82 | 3.32 |
| Day 2 | 1 | 1.31 | 3.9 | 3.65 |
|  | 2 | 1.34 | 3.81 | 3.56 |
|  | 3 | 1.31 | 3.77 | 3.58 |
|  | 4 | 1.27 | 3.84 | 3.64 |
|  | 5 | 1.35 | 3.82 | 3.51 |
| Day 3 | 1 | 1.33 | 3.89 | 2.95 |
|  | 2 | 1.35 | 3.82 | 3.56 |
|  | 3 | 1.33 | 3.82 | 3.38 |
|  | 4 | 1.37 | 3.87 | 3.42 |
|  | 5 | 1.3 | 3.69 | 3.42 |
| Day 4 | 1 | 1.41 | 3.98 | 3.61 |
|  | 2 | 1.43 | 3.9 | 3.52 |
|  | 3 | 1.41 | 3.93 | 3.42 |
|  | 4 | 1.4 | 3.7 | 3.54 |
|  | 5 | 1.41 | 3.81 | 3.5 |
| Day 5 | 1 | 1.36 | 4.07 | 3.57 |
|  | 2 | 1.37 | 4 | 3.52 |
|  | 3 | 1.36 | 4.01 | 3.59 |
|  | 4 | 1.34 | 3.98 | 3.48 |
|  | 5 | 1.35 | 4.01 | 3.61 |

Supplementary Table S1. Precision vertification with the MAGLUMI HEV IgM assay.

QC, quality control.
