# Supplementary material for: Evaluation of the analytical performance of the MAGLUMI HEV IgM and IgG assays for automated detection of HEV antibodies and comparison with the microplate Wantai assay
Source: Virol J. 2026 May 8;23:167. doi: 10.1186/s12985-026-03187-1 (PMC13321755; doi:10.1186/s12985-026-03187-1)
Supplement: Supplementary file 2 — Supplementary Material 2. [file 12985_2026_3187_MOESM2_ESM.docx]

| Days | Replicates | Test Results | | |
| --- | --- | --- | --- | --- |
|  |  | Sample 1  ( low concentration) | Sample 2  ( medium concentration) | Sample 3  (QC) |
| Day 1 | 1 | 1.78 | 10.3 | 3.98 |
|  | 2 | 1.77 | 9.49 | 3.8 |
|  | 3 | 1.88 | 9.87 | 3.72 |
|  | 4 | 1.95 | 9.58 | 3.68 |
|  | 5 | 1.8 | 9.71 | 3.61 |
| Day 2 | 1 | 1.84 | 9.75 | 3.87 |
|  | 2 | 1.76 | 9.94 | 3.99 |
|  | 3 | 1.83 | 10.5 | 3.67 |
|  | 4 | 1.85 | 10.4 | 3.75 |
|  | 5 | 1.77 | 9.4 | 4.08 |
| Day 3 | 1 | 1.79 | 10.3 | 3.97 |
|  | 2 | 1.78 | 10.2 | 3.87 |
|  | 3 | 1.91 | 9.96 | 3.99 |
|  | 4 | 1.83 | 10.2 | 4.15 |
|  | 5 | 1.84 | 10.1 | 3.67 |
| Day 4 | 1 | 1.79 | 10.5 | 3.97 |
|  | 2 | 1.79 | 10.4 | 3.88 |
|  | 3 | 1.88 | 10.4 | 3.91 |
|  | 4 | 1.83 | 9.98 | 3.41 |
|  | 5 | 1.77 | 10.1 | 4.01 |
| Day 5 | 1 | 1.81 | 10.6 | 4.25 |
|  | 2 | 1.79 | 10.8 | 4 |
|  | 3 | 1.78 | 9.46 | 4.11 |
|  | 4 | 1.8 | 10.5 | 4.14 |
|  | 5 | 1.79 | 9.98 | 4.16 |

Supplementary Table S2. Precision vertification with the MAGLUMI HEV IgG assay.

QC, quality control.
