# Supplementary material for: Evaluation of the analytical performance of the MAGLUMI HEV IgM and IgG assays for automated detection of HEV antibodies and comparison with the microplate Wantai assay
Source: Virol J. 2026 May 8;23:167. doi: 10.1186/s12985-026-03187-1 (PMC13321755; doi:10.1186/s12985-026-03187-1)
Supplement: Supplementary file 3 — Supplementary Material 3. [file 12985_2026_3187_MOESM3_ESM.docx]

| Characteristics | MAGLUMI HEV IgM evaluation (n=277) | MAGLUMI HEV IgG evaluation (n=326) |
| --- | --- | --- |
| Female | 91 (32.9%) | 104 (31.9%) |
| Male | 186 (67.1%) | 222 (68.1%) |
| Age (mean ± SD) | 55.1 ± 13.0 | (55.2 ± 12.7) |
| Sign and symptoms |  |  |
| Yellow sclera, staining of the skin, and dark-colored urine | 213 (76.9%) | 254 (77.9%) |
| Jaundice | 43 (15.5%) | 26 (8.0%) |
| Liver symptoms (hepatitis, liver injury, liver damage, liver failure, liver cirrhosis, percussion pain in the liver area, etc.) | 138 (49.8%) | 155 (47.5%) |
| General discomfort such as fever, malaise and fatigue | 212 (76.5%) | 245 (75.2%) |
| Digestive tract symptoms (nausea, vomiting, poor appetite, loss of appetite, abdominal pain, abdominal distension, etc.) | 225 (81.2%) | 258 (79.1%) |
| Final diagnosis (defined by RT-PCR) |  |  |
| viremic phase | 39 (14.1%) | 0 (0%) |
| post-viremic phase | 3 (1.1%) | 0 (0%) |
| uninfected | 8 (2.9%) | 0 (0%) |
| unknown | 227 (81.9%) | 326 (100%) |

Supplementary Table S3. Baseline characteristics of suspected HEV infection patients (n (%)).

HEV, hepatitis E virus; SD, standard deviation; RT-PCR, real-time polymerase chain reaction.
