# Supplementary material for: Evaluation of the analytical performance of the MAGLUMI HEV IgM and IgG assays for automated detection of HEV antibodies and comparison with the microplate Wantai assay
Source: Virol J. 2026 May 8;23:167. doi: 10.1186/s12985-026-03187-1 (PMC13321755; doi:10.1186/s12985-026-03187-1)
Supplement: Supplementary file 4 — Supplementary Material 4. [file 12985_2026_3187_MOESM4_ESM.docx]

|  | Test results | |
| --- | --- | --- |
| Sample ID | MAGLUMI HEV IgM  (AU/mL) | Wantai HEV IgM  (A value) |
| M028 | 6.600  (positive) | 0.001  (negative) |
| M044 | 2.900  (positive) | 0.025  (negative) |
| M171 | 0.768  (negative) | 0.927  (positive) |

Supplementary Table S4. Detailed results for the discrepant results of HEV IgM assays.

HEV, hepatitis E virus.
