# Supplementary material for: Evaluation of the analytical performance of the MAGLUMI HEV IgM and IgG assays for automated detection of HEV antibodies and comparison with the microplate Wantai assay
Source: Virol J. 2026 May 8;23:167. doi: 10.1186/s12985-026-03187-1 (PMC13321755; doi:10.1186/s12985-026-03187-1)
Supplement: Supplementary file 5 — Supplementary Material 5. [file 12985_2026_3187_MOESM5_ESM.docx]

|  | Test results | |
| --- | --- | --- |
| Sample ID | MAGLUMI HEV IgG  (AU/mL) | Wantai HEV IgG  (A value) |
| G018 | 0.154  (negative) | 0.760  (positive) |
| G035 | 0.593  (negative) | 0.326  (positive) |
| G036 | 40.700  (positive) | 0.001  (negative) |
| G049 | 14.300  (positive) | 0.003  (negative) |
| G055 | 1.320  (positive) | 0.001  (negative) |
| G100 | 4.150  (positive) | 0.030  (negative) |
| G113 | 18.600  (positive) | 0.009  (negative) |
| G132 | 0.151  (negative) | 0.365  (positive) |
| G133 | 0.840  (negative) | 3.338  (positive) |
| G136 | 0.210  (negative) | 0.528  (positive) |
| G150 | 8.560  (positive) | 0.003  (negative) |
| G154 | 0.437  (negative) | 0.482  (positive) |
| G155 | 0.790  (negative) | 0.749  (positive) |
| G161 | 0.103  (negative) | 0.955  (positive) |
| G229 | 0.141  (negative) | 0.865  (positive) |
| G274 | 0.080  (negative) | 0.199  (positive) |

Supplementary Table S5. Detailed results for the discrepant results of HEV IgG assays.

HEV, hepatitis E virus.
