# Supplementary material for: Evaluation of the analytical performance of the MAGLUMI HEV IgM and IgG assays for automated detection of HEV antibodies and comparison with the microplate Wantai assay
Source: Virol J. 2026 May 8;23:167. doi: 10.1186/s12985-026-03187-1 (PMC13321755; doi:10.1186/s12985-026-03187-1)
Supplement: Supplementary file 6 — Supplementary Material 6. [file 12985_2026_3187_MOESM6_ESM.docx]

|  | MAGLUMI HEV IgM | |
| --- | --- | --- |
| HEV infection status | Positive | Negative |
| viremic phase (IgM+RNA+) | 39 | 0 |
| post-viremic phase (IgM+RNA-) | 3 | 0 |
| uninfected (IgM-RNA-) | 0 | 94 |
| sensitivity | 100.00% (95% CI: 91.62–100.00%) | |
| specificity | 100.00% (95% CI: 96.07–100.00%) | |

Supplementary Table S6. Sensitivity and specificity of the MAGLUMI HEV IgM assay in relation to the infectious profile.

HEV, hepatitis E virus; CI, Confidence Interval.
